# Broad-spectrum XX and XY gonadal dysgenesis in patients with a homozygous L193S variant in *PPP2R3C*

**DOI:** 10.1530/EJE-21-0910

**Published:** 2021-10-28

**Authors:** Dilek Cicek, Nick Warr, Gozde Yesil, Hatice Kocak Eker, Firdevs Bas, Sukran Poyrazoglu, Feyza Darendeliler, Gul Direk, Nihal Hatipoglu, Mehmet Eltan, Zehra Yavas Abali, Busra Gurpinar Tosun, Sare Betul Kaygusuz, Tuba Seven Menevse, Didem Helvacioglu, Serap Turan, Abdullah Bereket, Richard Reeves, Michelle Simon, Matthew Mackenzie, Lydia Teboul, Andy Greenfield, Tulay Guran

**Affiliations:** 1 Department of Paediatric Endocrinology and Diabetes, Erciyes University, School of Medicine, Kayseri, Turkey; 2 Mammalian Genetics Unit, Medical Research Council Harwell Institute, Harwell, Oxfordshire, UK; 3 Department of Medical Genetics, Istanbul University, School of Medicine, Istanbul, Turkey; 4 Department of Medical Genetics, Konya Training and Research Hospital, Konya, Turkey; 5 Department of Pediatric Endocrinology and Diabetes, Istanbul University, School of Medicine, Istanbul, Turkey; 6 Department of Paediatric Endocrinology and Diabetes, Marmara University, School of Medicine, Istanbul, Turkey; 7 Mary Lyon Centre, Medical Research Council Harwell Institute, Harwell, Oxfordshire, UK

## Abstract

**Context:**

Homozygous and heterozygous variants in *PPP2R3C* are associated with syndromic 46,XY complete gonadal dysgenesis (Myo-Ectodermo-Gonadal Dysgenesis (MEGD) syndrome), and impaired spermatogenesis, respectively. This study expands the role of *PPP2R3C* in the aetiology of gonadal dysgenesis (GD).

**Method:**

We sequenced the *PPP2R3C* gene in four new patients from three unrelated families. The clinical, laboratory, and molecular characteristics were investigated. We have also determined the requirement for *Ppp2r3c* in mice (C57BL6/N) using CRISPR/Cas9 genome editing.

**Results:**

A homozygous c.578T>C (p.L193S) *PPP2R3C* variant was identified in one 46,XX girl with primary gonadal insufficiency, two girls with 46,XY complete GD, and one undervirilised boy with 46,XY partial GD. The patients with complete GD had low gonadal and adrenal androgens, low anti-Müllerian hormone, and high follicle-stimulating hormone and luteinizing hormone concentrations. All patients manifested characteristic features of MEGD syndrome. Heterozygous *Ppp2r3c* knockout mice appeared overtly normal and fertile. Inspection of homozygous embryos at 14.5, 9.5, and 8.5 days *post coitum*(dpc) revealed evidence of dead embryos. We conclude that loss of function of *Ppp2r3c* is not compatible with viability in mice and results in embryonic death from 7.5 dpc or earlier.

**Conclusion:**

Our data indicate the essential roles for *PPP2R3C* in mouse and human development. Germline homozygous variants in human *PPP2R3C* are associated with distinctive syndromic GD of varying severity in both 46,XY and 46,XX individuals.

## Introduction

Gonadal dysgenesis (GD) encompasses a variety of rare conditions that cause impaired development of the gonads, that is, the testes or the ovaries (1). The average prevalence of GD is 1–9 cases per 100 000 live-born (2), which can be classified as either complete (CGD) or partial (PGD) depending on gonadal morphology. All cases of 46,XX GD (XX-GD) and XY-CGD patients have a completely female phenotype, whereas in XY-PGD, the external genitalia phenotype depends on the degree of testicular function. Although the underlying aetiology remains unknown in most cases, several genes have been implicated in XY and XX-GD (1, 3, 4, 5).

We have recently reported homozygous variants in *PPP2R3C* in four patients with syndromic XY-CGD, namely MEGD syndrome (Myo-Ectodermo-Gonadal Dysgenesis) (6). Besides XY-CGD, a number of extra-gonadal syndromic features, including typical facial gestalt, low birth weight, myopathy, rod and cone dystrophy, anal atresia, omphalocele, sensorineural hearing loss, dry and scaly skin, skeletal abnormalities, renal agenesis, and neuromotor delay characterize MEGD syndrome. Here, we describe one 46,XX girl with primary gonadal insufficiency and one undervirilised boy with XY-PGD, besides two new patients with XY-CGD. Our case series expands the spectrum of GD in MEGD syndrome and establishes the emerging role of *PPP2R3C* in gonad development, in both 46,XX and 46,XY individuals. We have also determined the requirement for *Ppp2r3c* in mice using CRISPR/Cas9 genome editing and show that it is an essential gene.

## Patients and methods

### Clinical studies

All clinical investigations and genetic analyses were performed according to the guidelines of the Declaration of Helsinki and with written consent of the families. The Ethical Committee of Marmara University, Istanbul, Turkey approved the study (09.2017.471). Written informed consent for publication of their clinical details and/or clinical images was obtained from the parents of the patients.

Four patients from three independent consanguineous families were evaluated for a GD with common syndromic features compatible with MEDG syndrome. Detailed clinical, laboratory, and molecular characteristics of the patients and families are described.

### Sanger sequencing

All the coding exons and intron–exon boundaries of the *PPP2R3C* gene were amplified by the PCR (6). Sequence reactions were run on an ABI Prism 3130xl DNA Sequencer and analysed by Seqscape sequencing analysis software, version 2.7 (Applied Biosystems).

### Bioinformatics

The Heatmap was generated using pheatmap v1.0.12. Cells were ordered using cluster annotations obtained from https://github.com/IStevant/XX-XY-mouse-gonad-scRNA-seq/tree/master/data.

### Mouse strains and CRISPR/Cas9 genome editing

All experiments were performed on the C57BL/6N genetic background, which like C57BL/6J is sensitised to disruptions to testis determination (7, 8). In order to generate a predicted null allele of *Ppp2r3c*, CRISPR/Cas9 genome editing was used to introduce a deletion encompassing the whole of exon 5 (ENSMUSE00000435370). Briefly, a pair of single-guide RNAs, which flank exon 5, were electroporated into one-cell C57BL/6Ntac embryos along with Cas9 protein, as previously described (9). gRNA protospacer sequences were 5’-CGTGTTGTATTAGGATTGCA-3’ and 5’-TGCATATACCACCCATACGA-3’. A founder was identified harbouring a 1057-bp deletion that removes the whole of *Ppp2r3c* exon 5, following screening and quality assessment as previously described (10). Transmission of this deletion allele was confirmed by breeding. Genotyping was based on a three-primer strategy: forward primer 5’-ACGAAGAGTTGCAGGTACGA-3’ and reverse primers 5’-GGCAGGGCTAGGATCTAACC-3’ and 5’-TCTATGAAGAAGAGGGTGGCT-3’. The WT allele gives a product size of 270 bp, whilst the deletion allele gives a product size of 390 bp.

### Embryo generation and wholemount *in situ* hybridisation (WMISH)

Timed mates between heterozygous animals were performed in order to generate homozygous and heterozygous embryos. Noon on the day that a copulatory plug was observed was counted as 0.5 days *post coitum* (dpc). Mice were sacrificed in a humane fashion by dislocation of the neck; embryos were decapitated in ice-cold, PBS. All animal experimentation was approved by the Animal Welfare and Ethical Review Body at MRC Harwell Institute. Mice were bred under license from the UK Home Office (PPL 70/8898 and PP5230673) and used according to the Animals (Scientific Procedures) Act, 1986. WMISH of embryonic gonads and the use of probes for the genes *Sox9* and *Stra8* have been described previously (11, 12).

## Results

### Case reports

**Figure 1:**
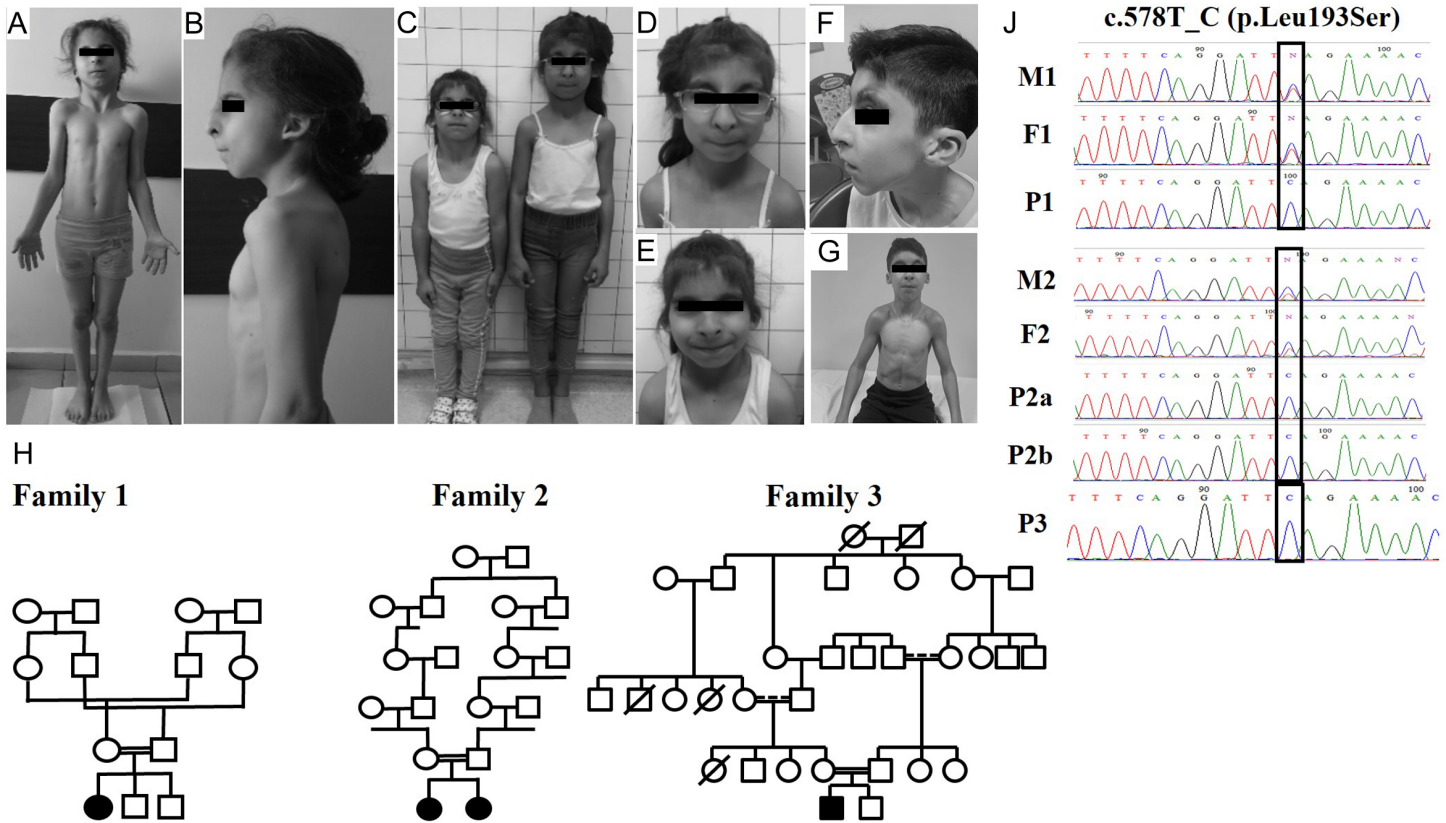
Clinical and molecular characteristics of patients with MEGD syndrome. Phenotypical features (A, B, C, D, E, F, and G), pedigrees (H), and sequencing chromatograms (J) of patients and parents. A full colour version of this figure is available at https://doi.org/10.1530/EJE-21-0910.

One male and three female patients were evaluated for syndromic GD. They had common facial characteristics of MEGD syndrome, including flat face, flat vertex, unusual scalp hair whorls, unruly scalp hair, frontal upsweep, arched and sparse eyebrows, bilateral epicanthal folds, thin lips, long and smooth philtrum, and hypodontia. All had hypoplastic ala nasi and beaked nose with ‘squashed down’ appearance. The patients also had low set and posteriorly rotated ears, overfolded helix, and narrowed and elongated intertragic notch with or without sinus just under the tip of the intertragal angle (Fig. 1A, B, C, D, E, F and G). Short and broad hands, a horizontal single crease, cutaneous syndactyly (interphalangeal webbing) in hands were present in all patients. All patients had myopathy and significant muscular build with thick and stiff muscles particularly in the neck. They had significantly delayed bone ages. Additionally, they had some common growth, skin, gastrointestinal, renal and cardiac abnormalities and neuromotor delay and mental retardation. Further clinical characteristics of patients were as follows:

**Patient 1 (P1).** The baby (46,XX) was born at 38 weeks gestation by caesarean section; birth weight was 1900 g (−3.3 SDS(standard deviation score)). Parents were consanguineous (Fig. 1H). She had renal agenesis on the left side and epilepsy. At 9 years and 4 months, she was assessed for short stature. Parents were consanguineous. She weighed 16.1 kg (−4.1 SDS) and was 110.2 cm tall (−4.1 SDS). Physical examination showed several dysmorphic features (Fig. 1A and  B) and normal female external genitalia. She was prepubertal. She had mildly poor school performance and some visual impairment due to rod–cone dystrophy. She had high serum creatine kinase concentrations. Her serum insulin-like growth factor 1 and insulin-like growth factor binding protein 3 concentrations and growth hormone stimulation tests were normal. Bone age was 6 years and 10 months. Her gonadal and adrenal function tests are presented in Table 1. She had normal early morning cortisol and low DHEAS. High gonadotrophin and low anti-Müllerian hormone (AMH) concentrations suggested GD. Pelvic ultrasound revealed a prepubertal uterus, but gonads could not be visualised.

**Table 1:** Gonadal and adrenal function test results of patients with MEGD syndrome.

Hormone	P1 (9^5/12^)		P2a (7^11/12^)		P2b (6^1/12^)		P3 (10^5/12^)	
	Value	Normal range	Value	Normal range	Value	Normal range	Value	Normal range
Karyotype	46, XX		46, XY		46, XY		46, XY	
FSH (mIU/mL)	41.1	1.79–10.9	44.59	1.9–12.8	43.1	3.85–8.78	11.8	1.5–12.40
LH (mIU/mL)	9.07	1–11	1.81	<0.3–6.3	1.7	<0.3–6.3	2.8	1.7–8.6
Testosterone (ng/mL)	<0.07	2.4–9.5	<0.07	2.4–9.5	<0.07	2.4–9.5	1.3	2.4–9.5
Estradiol (pg/mL)	<20	27–122	<20	27–122	<20	27–122	NA	
AMH (ng/mL)	0.01	0.00–8.8	0.00	0.00–8.8	0.00	0.00–8.8	18.8	38.2–332
Cortisol (µg/dL)	9.8	5–21	16.1	5–21	11.8	5–21	12.8	5–21
DHEAS (ng/mL)	49	80–560	17.5	35–430	15	35–430	26	80–560
Androstenedione (ng/mL)	0.81	0.6–3.1	<0.3	0.3–2	<0.3	0.3–2	NA	

AMH, anti-Müllerian hormone; FSH, follicle-stimulating hormone; LH, luteinizing hormone.
**Patient 2a (P2a).** The baby (46,XY, SRY(+)) was evaluated for dysmorphic features. She was born at 36.5 weeks gestation by vaginal delivery; her birth weight was 2200 g (−2.2 SDS). Parents were consanguineous (Fig. 1H). At 7 years and 11 months, she weighed 21.2 kg (−0.8 SDS) and was 110.2 cm tall (−0.9 SDS). Physical examination showed several dysmorphic features (Fig. 1A and  B) and normal female external genitalia. She was prepubertal. Bone age was 4 years. She had developmental delay, myopia, and amblyopia. She had normal early morning cortisol, low concentrations of adrenal and gonadal androgens, low AMH, and high gonadotrophins (Table 1), suggesting CGD.
**Patient 2b (P2b).** The younger sister of Patient 2a (46,XY, SRY(+)) presented to the hospital for similar dysmorphic features. She was born at 37 weeks gestation by vaginal delivery; her birth weight was 2500 g (−1.96 SDS). At 6 years and 1 months, she weighed 16.7 kg (−2.07 SDS) and was 107.1 cm tall (−1.98 SDS). Physical examination showed several dysmorphic features (Fig. 1A and  B) and normal female external genitalia. She was prepubertal. She had developmental delay, myopia, amblyopia, developmental hip dysplasia, and kyphosis. Her adrenal and gonadal function tests were similar to her elder sister, consistent with CGD (Table 1).
**Patient 3 (P3).** The baby (46,XY, SRY(+)) was evaluated for ambiguous genitalia. He was born at 40 weeks gestation by vaginal delivery; his birth weight was 3190 g (−0.72 SDS). Parents were consanguineous (Fig. 1H). He had micropenis (stretched penile length; 1.5 cm), left cryptorchidism, penoscrotal hypospadias with chordee, and bifid scrotum. Histopathologic examination of biopsy of left gonad revealed immature testis at 8 months of age. He had a 3-day human chorionic gonadotrophin test at 5 months of age, which showed adequate testosterone response (from 0.23 to 6.4 ng/mL). He was operated for cryptorchidism and hypospadias at 3 and 4 years of age, respectively. He had agenesis of ductus deferens on right side. He showed normal neuromotor development. At 10.5 years, he weighed 27.2 kg (−1.4 SDS) and was 133.7 cm tall (−1.13 SDS). Physical examination showed dysmorphic features compatible with MEGD syndrome (Fig. 1F and  G). His testicular volumes were 3 cc bilaterally, stretched penile length was 4 cm, and pubic hair was Tanner stage 1. He had low DHEAS adrenal and gonadal function tests and low AMH concentrations, suggesting PGD (Table 1).

G-banding karyotyping was used to confirm the chromosomal analysis of the patients and revealed no large genomic rearrangements. We subsequently identified a homozygous c.578 T>C (p.Leu193Ser) variant in *PPP2R3C* in all patients using Sanger sequencing. In all cases, parents were heterozygous carriers of the variant (Fig. 1J). The p.Leu193Ser variant in *PPP2R3C* (rs1566411552) has previously been described in another Turkish family. There was no known relationship between these families.

### Generation and characterisation of *Ppp2r3C* loss-of-function mice using CRISPR/Cas9

**Figure 2:**
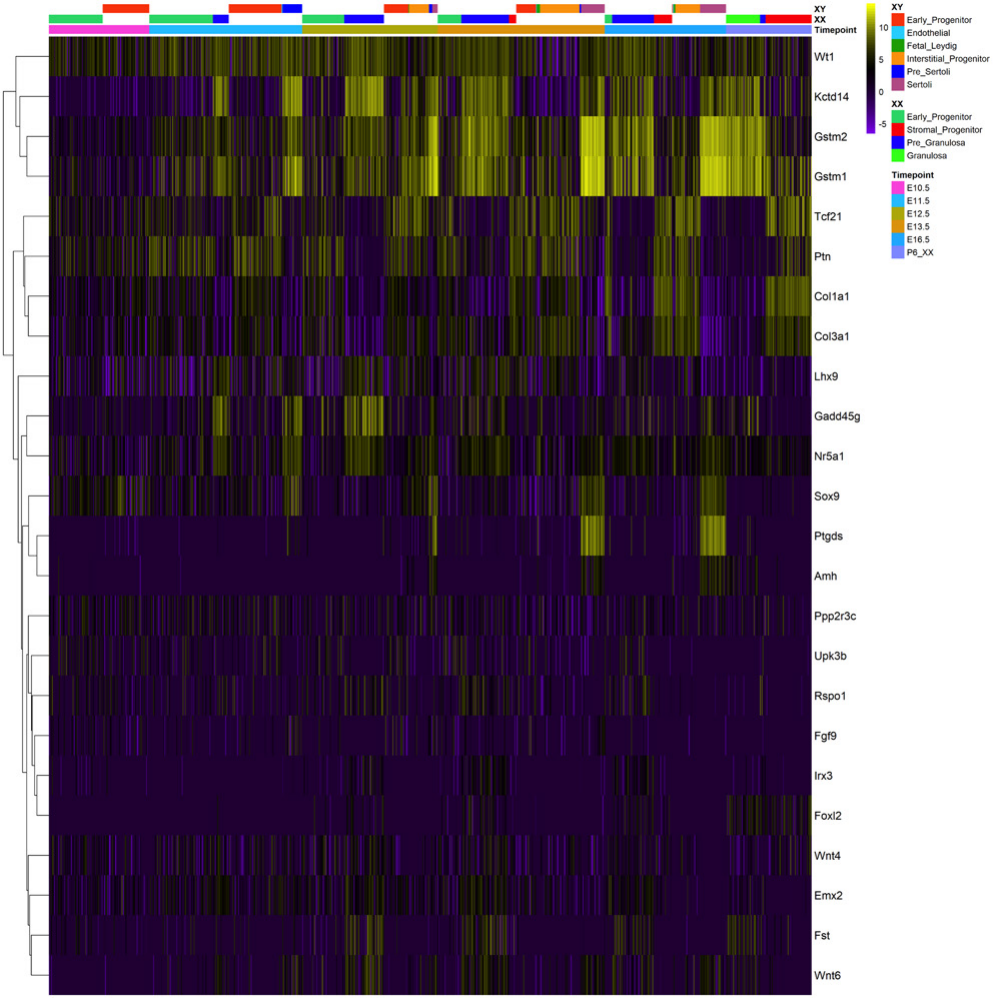
Analysis of single-cell RNAseq data reveals widespread *Ppp2r3c* expression in multiple gonadal cell lineages. Heatmap of scRNA-Seq expression in the mouse gonad at multiple time-points (10.5–16.5 dpc and P6 in XX mice). Single cells were ordered using cluster annotations obtained from https://doi.org/10.1530/EJE-21-0910.

We assessed the candidature of *PPP2R3C* as a mammalian testis-determining gene by examining *Ppp2r3c* expression in the developing mouse gonad during the sex-determining period (10.5–16.5 dpc). Using a published single-cell RNA sequencing (scRNAseq) dataset of XX and XY mouse gonad development (13), we identified expression in the majority of gonadal cell lineages, including *Tcf21*+ gonadal progenitors at 11.5 dpc and *Sox9*+ and *Fst*+ supporting cells in XY and XX gonads, respectively (Fig. 2). There was no evidence of any sexual dimorphism in levels of expression. Low-level, widespread expression of *Ppp2r3c* during this period in a number of cell lineages that contribute to the gonadal supporting cells (14) was consistent with a sex-determining function.

To directly assess whether PPP2R3C is required for sex determination in mice, a loss-of-function allele was generated using CRISPR/Cas9-mediated genome editing. The mouse *Ppp2r3c* gene has 14 exons (ENSMUST00000021410.10). Loss of exon 5 is predicted to result in a premature frameshift mutation following splicing of exon 4 to exon 6 and therefore a severely truncated version of the protein. We used a pair of single-guide RNAs to direct Cas9-mediated cutting to regions on either side of exon 5 (see materials and methods). Founder mice were analysed for deletion events in the vicinity of exon 5, and a founder was identified harbouring a 1057-bp deletion, encompassing the whole of exon 5 and flanking regions. Transmission of this allele, which we term Δ1057, was confirmed by breeding of the founder.

### Analysis of gonad development in embryos lacking functional PPP2R3C

**Figure 3:**
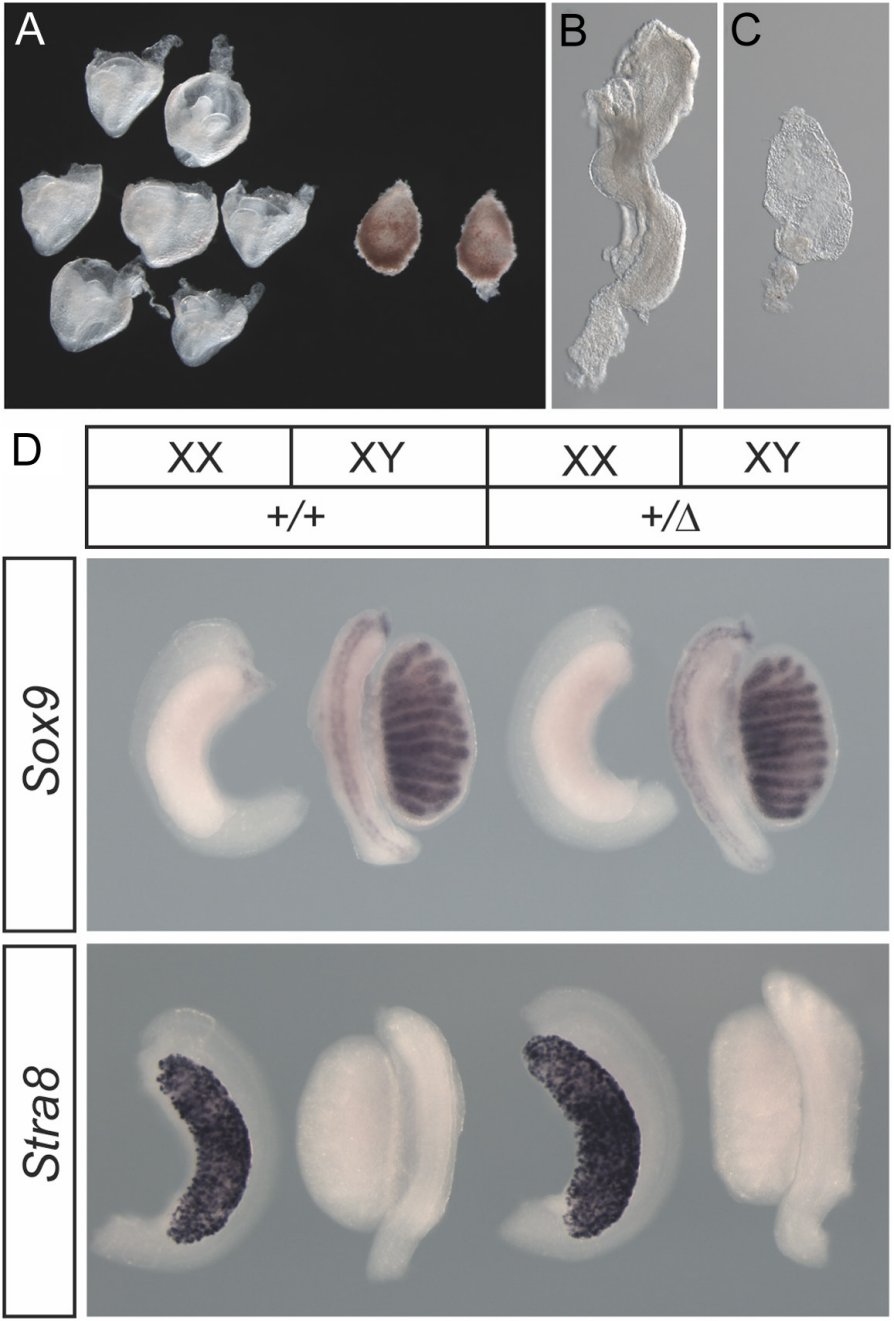
Loss of *Ppp2r3c* causes embryonic death, but heterozygous mutants have normal foetal gonads. (A) A litter from a heterozygote inter-cross dissected at 8.5 dpc, showing approximately one-quarter are undeveloped. The small, most often empty, Reichert’s membranes genotyped as homozygous for the *Ppp2r3c* Δ1057 deletion; (B) carefully dissected control (+/+) embryo at 8.5 dpc compared to (C) a malformed homozygous embryo remnant; (D) wholemount *in situ* hybridisation staining of heterozygous (+/Δ) gonads of both sexes at 14.5 dpc. Probes used are indicated. *Sox9* marks Sertoli cells in the testis cords of WT (+/+) XY gonads, whilst *Stra8* marks germ cell entering meiosis in the WT XX gonad. No indications of gonadal sex reversal were observed in the heterozygous (+/Δ) gonads, which were morphologically identical to controls (+/+). A full colour version of this figure is available at https://doi.org/10.1530/EJE-21-0910.

Heterozygous (+/Δ1057) male and female adult mice were overtly normal and fertile. In order to examine gonad development in homozygous embryos, we inter-crossed heterozygous animals. We performed uterine openings at 14.5 dpc and genotyped all embryos (Fig. 3 and Table 2). No homozygous (Δ/Δ) embryos were detected at this stage, and this absence was statistically significant (Table 2). Dissections at 9.5 dpc revealed no live homozygous embryos, but dead and dying material was identified and genotyped as homozygous (Fig. 3B and  C). At 8.5 dpc, empty Reichert’s membranes were identified, as were embryo remnants, which were genotyped as homozygous (Fig. 3A). These data suggest that homozygous embryos are absent or dying/dead at 8.5 dpc, and this loss of viability may begin even earlier in gestation. Therefore, *Ppp2r3c* is an essential gene for early mouse development. In the absence of homozygous embryos at the relevant stages, we examined gonad development in heterozygous embryos and observed no clear abnormalities at 14.5 dpc (Fig. 3D). XY +/Δ1057 gonads had testis cord development characterised by robust *Sox9* expression in Sertoli cells, as in WT (+/+) embryos (Fig. 3D). They also completely lacked *Stra8* expression, a marker of germ cells entering meiosis that is expressed in WT XX gonads at the same stage, suggesting that no ovarian tissue is present in them. *Stra8* and *Sox9* expression was also normal in XX +/Δ1057 gonads (Fig. 3D). These data suggest no overt sex reversal phenotype is detectable in the developing gonads of XY or XX heterozygous animals.

**Table 2:** Absence of viable homozygous (Δ/Δ) embryos at 14.5, 9.5, and 8.5 days *post coitum* (dpc).

	+/+	+/Δ	Δ/Δ	Total	*P*
14.5 dpc					
Normal embryos	10 (6, 4)	17 (7, 10)	–	27	***
9.5 dpc					
Normal embryos	5	7	–	12	*
Dead/dying	–	1	2	3	
8.5 dpc					
Normal embryos	8	13	–	21	**
Embryo remnant	–	–	1	1	
Empty Reichert’s	–	1	6	7	

*P ≤ 0.05; **P ≤ 0.005; ***P ≤ 0.001 (Student’s *t*-test).

## Discussion

Our case series refines the GD spectrum of MEGD syndrome, describing four new patients with a homozygous c.578T>C (p.L193S) variant in the *PPP2R3C* gene and the functional impact of *Ppp2r3c* in the mouse model. This showed that variants in *PPP2R3C* are associated with GD of variable severity, both in 46,XX and 46,XY. Four of our cases presented with different phenotypes: 46,XX GD (P1), 46,XY CGD (P2a and 2b), and a 46,XY PGD (P3) phenotype.

Human sexual development starts at the 5th–6th embryonic weeks. The genital ridges are converted to bipotential gonads, which subsequently differentiate into ovaries or testes. In the XY foetus, expression of testis-determining *SRY* triggers upregulation of *SOX9* expression*,* leading to testis formation (15). On the other hand, in the absence of *SRY*, the WNT/β-catenin signalling opposes testis determination and directs a female-specific molecular cascade and ovary formation (16). These pathways are regulated by a network of genes controlled by various transcriptional factors operating in a delicate equilibrium. These core, and mutually antagonistic, sex-determining gene regulatory networks are also conserved in mice (14, 17). Disturbances in these early developmental mechanisms result in GD in 46,XX and 46,XY individuals, causing a broad spectrum of clinical phenotypes ranging from primary amenorrhea to ambiguous genitalia and complete gonadal sex reversal.

Molecular defects causing XX-GD include *RSPO1*, *LARS2*, *HSD17B4*, *HARS2*, *TWNK*, *ERAL1*, and *CLPP*, while *ARX*, *ATRX*, *DHH*, *GATA4*, *HHAT*, *SOX9*, *WT1*, and *ZFPM2* gene defects are associated with syndromic XY-GD (18). Extra-gonadal phenotypes of these syndromes include malformations of various organs including CNS and brain, bone, heart, kidney, etc.

We have recently reported four patients from four unrelated consanguineous Turkish families with syndromic XY-CGD (MEGD syndrome) due to homozygous *PPP2R3C* variants, confirmed by hormone profile, imaging techniques, and histopathology. Decreased SOX9-phospho-protein expression in the dysgenetic gonads of patients with homozygous *PPP2R3C* variants suggested impaired SOX9 signalling underlying the pathogenesis of GD in this condition (6).

The essential role of post-transcriptional phoshoregulatory pathways in gonadal development has emerged by identification of rare human phenotypes with genetically proven defects. Hughes *et al*. recently described 12 individuals with holoprosencephaly spectrum phenotypes and urogenital malformations, including variant sex development, due to *PPP1R12A* variants, which encodes protein phosphatase 1, regulatory subunit 12a (19). They have shown high expression of PPP1R12A in the developing reproductive system and brain structures by mouse immunostaining studies. 46,XY individuals in that cohort exhibited abnormalities of sex development, ranging from mild hypospadias and micropenis to complete female genitalia and streak gonads with uterus. Patients in that cohort have some overlapping clinical characteristics with MEGD syndrome patients, including XY-CGD and XY-PGD, kyphoscoliosis, developmental delay, flattened facial profile, rod–cone dystrophy, and structural renal problems. Although interactome analysis using two databases (https://string-db.org/, https://inbio-discover.com/) did not support the functional association of* PPP2R3C* and* PPP1R12A*, the overlapping phenotypes of syndromic GD due to the variants in these two genes suggest the potential role of other phosphoprotein phosphatases in gonadal development.

We proposed that the variants in *PPP2R3C* upregulate the catalytic function of PP2A and increase the dephosphorylation of active SOX9-phosphoprotein, which impairs SOX9 and results in the pathogenesis of XY-GD (6). PP2A also regulates the WNT pathway at multiple levels. PP2A is considered a negative regulator of the WNT pathway since PP2A inhibition results in enhancement of ß-catenin-dependent transcription. PP2A also binds to β-catenin and suppresses its phosphorylation, thereby increasing the total β-catenin availability (20). We suggest that variants in *PPP2R3C* similarly enhance the function of PP2A and suppress WNT/β-catenin signalling, which subsequently impairs ovarian development resulting in XX-GD. These proposed mechanisms may explain XY and XX-GD seen in our patients.

The c.578T>C (p.L193S) variant identified in our patients was found neither in 200 ethnically matched in-house Turkish exomes and the Turkish whole-exome database nor in 20 in-house exomes from other Turkish 46, XY DSD patients. This variant was not seen in either GnomAD, ExAC, 1000 Genomes, 6500ESP, or the Turkish in-house databases. PolyPhen-2 and scale-invariant feature transform (SIFT) predict c.578 T>C (p.L193S) to be probably damaging and deleterious with scores of 0.985 and 0, respectively. Mutation Taster predicts this variant to be disease causing. The PhyloP and PhastCons scores, which are 4.719 and 1 for c.578T>C variant, show that this mutated region is slowly evolving and conserved among species. Identification of same variant in unrelated families with different geographical origins, and the identification of the same variation in our previous patients, suggests a founder effect of p.L193S in *PPP2R3C* in the Turkish population.

In order to determine the role of PPP2R3C in mammalian reproductive biology, in particular gonadal sex determination, we generated mice (C57BL6/N) lacking functional *Ppp2r3c* by using CRISPR/Cas9 genome editing to delete a 1057-bp segment encompassing exon 5. Heterozygotes for the deletion allele appeared overtly normal and fertile. Timed mates between heterozygous animals were used to generate homozygotes at 14.5 dpc in order to allow inspection of the foetal gonads after the sex-determining period. However, genotyping revealed that no homozygous animals were detected at this stage. Inspection of embryos at earlier stages (9.5 and 8.5 dpc) revealed evidence of dead or dying embryonic tissue. Genotyping indicated that this material was derived exclusively from homozygous embryos. We conclude that loss of function of *Ppp2r3c* is not compatible with viability in mice and results in embryonic death from 7.5 dpc or earlier. Conditional gene targeting will be required to determine the role of PPP2R3C in the developing mouse gonad.

In summary, the four individuals reported here illustrate the association of *PPP2R3C* variants with GD spectrum phenotypes and multiple malformations in MEGD syndrome. In the mouse model, studies of *Ppp2r3c* demonstrate expression in a number of developing gonadal cell lineages important for sex determination and an essential role in development.

## Declaration of interest

The authors declare that there is no conflict of interest that could be perceived as prejudicing the impartiality of this study.

## Funding

This work has been supported by the Medical Research Council http://dx.doi.org/10.13039/501100000265 of Marmara University (Project Grant SAG-A-120418-0152). Research in AG’s laboratory was funded by the UK Medical Research Council http://dx.doi.org/10.13039/501100000265 through core funding (MC_U142684167) at the Mammalian Genetics Unit, MRC Harwell Institute.

## Author contribution statement

T G, A G, D C, and G Y designed the study. T G, D C, Z Y A, H K E, F B, G D, S P, F D, B G T, T S M, M E, D H, S B K, D H, N H, S T, and A B recruited and clinically characterised the patients. T G and G Y conducted Sanger sequencing and analysis of the results. T G, A G, G Y, F D, and A B prepared the draft manuscript. All authors contributed to the discussion of results, and edited and approved the final manuscript. N W examined development in control and *Ppp2r3c* mutant embryos; R R and M S analysed single-cell RNAseq data; M M and L T performed and analysed results from CRISPR/Cas9 genome editing experiments. D C, N W, A G, and T G: contributed equally.

## References

[1] Breehl L Caban O. Genetics, gonadal dysgenesis. In StatPearls. Treasure Island (FL): StatPearls Publishing LLC, 2021. 30969708

[2] Berglund A Johannsen TH Stochholm K Viuff MH Fedder J Main KM Gravholt CH. Incidence, prevalence, diagnostic delay, and clinical presentation of female 46,XY disorders of sex development. Journal of Clinical Endocrinology and Metabolism 2016 101 4532–4540. (10.1210/jc.2016-2248) 27603905

[3] Baetens D Verdin H De Baere E Cools M. Update on the genetics of differences of sex development (DSD). Best Practice and Research: Clinical Endocrinology and Metabolism 2019 33 101271. (10.1016/j.beem.2019.04.005) 31005504

[4] Eozenou C Gonen N Touzon MS Jorgensen A Yatsenko SA Fusee L Kamel AK Gellen B Guercio G Singh P Testis formation in XX individuals resulting from novel pathogenic variants in Wilms’ tumor 1 (WT1) gene. PNAS 2020 117 13680–13688. (10.1073/pnas.1921676117) 32493750 PMC7306989

[5] McElreavey K Jorgensen A Eozenou C Merel T Bignon-Topalovic J Tan DS Houzelstein D Buonocore F Warr N Kay RGG Pathogenic variants in the DEAH-box RNA helicase DHX37 are a frequent cause of 46,XY gonadal dysgenesis and 46,XY testicular regression syndrome. Genetics in Medicine 2020 22 150–159. (10.1038/s41436-019-0606-y) 31337883 PMC6944638

[6] Guran T Yesil G Turan S Atay Z Bozkurtlar E Aghayev A Gul S Tinay I Aru B Arslan S PPP2R3C gene variants cause syndromic 46,XY gonadal dysgenesis and impaired spermatogenesis in humans. European Journal of Endocrinology 2019 180 291–309. (10.1530/EJE-19-0067) 30893644

[7] Warr N Carre GA Siggers P Faleato JV Brixey R Pope M Bogani D Childers M Wells S Scudamore CL Gadd45γ and Map3k4 interactions regulate mouse testis determination via p38 MAPK-mediated control of Sry expression. Developmental Cell 2012 23 1020–1031. (10.1016/j.devcel.2012.09.016) 23102580 PMC3526779

[8] Livermore C Simon M Reeves R Stévant I Nef S Pope M Mallon AM Wells S Warr N Greenfield A. Protection against XY gonadal sex reversal by a variant region on mouse chromosome 13. Genetics 2020 214 467–477. (10.1534/genetics.119.302786) 31836612 PMC7017026

[9] Gertsenstein M Nutter LMJ. Production of knockout mouse lines with Cas9. Methods 2021 191 32–43. (10.1016/j.ymeth.2021.01.005) 33524495

[10] Owens DDG Caulder A Frontera V Harman JR Allan AJ Bucakci A Greder L Codner GF Hublitz P McHugh PJ Microhomologies are prevalent at Cas9-induced larger deletions. Nucleic Acids Research 2019 47 7402–7417. (10.1093/nar/gkz459) 31127293 PMC6698657

[11] Bogani D Siggers P Brixey R Warr N Beddow S Edwards J Williams D Wilhelm D Koopman P Flavell RA Loss of mitogen-activated protein kinase kinase kinase 4 (MAP3K4) reveals a requirement for MAPK signalling in mouse sex determination. PLoS Biology 2009 7 e1000196. (10.1371/journal.pbio.1000196) PMC2733150 19753101

[12] Carré GA Siggers P Xipolita M Brindle P Lutz B Wells S Greenfield A. Loss of p300 and CBP disrupts histone acetylation at the mouse Sry promoter and causes XY gonadal sex reversal. Human Molecular Genetics 2018 27 190–198. (10.1093/hmg/ddx398) 29145650 PMC5886154

[13] Stévant I Kühne F Greenfield A Chaboissier M-C Dermitzakis ET Nef S. Single-cell transcriptomics of the mouse gonadal soma reveals the establishment of sexual dimorphism in distinct cell lineages. bioRxiv 2018 410407. (10.1101/410407)

[14] Nef S Stevant I Greenfield A. Characterizing the bipotential mammalian gonad. In Sex Determination in Vertebrates. Current Topics in Developmental Biology, Vol. 132, pp. 167–194. Ed Capel B San Diego: Elsevier Academic Press Inc., 2019. (10.1016/bs.ctdb.2019.01.002) 30999975

[15] Lucas-Herald AK Bashamboo A. Gonadal development. Endocrine Development 2014 27 1–16. (10.1159/000363608) 25247640

[16] Parma P Radi O. Molecular mechanisms of sexual development. Sexual Development 2012 6 7–17. (10.1159/000332209) 22025195

[17] Greenfield A Understanding sex determination in the mouse: genetics, epigenetics and the story of mutual antagonisms. Journal of Genetics 2015 94 585–590. (10.1007/s12041-015-0565-2) 26690512

[18] Audi L Ahmed SF Krone N Cools M McElreavey K Holterhus PM Greenfield A Bashamboo A Hiort O Wudy SA GENETICS IN ENDOCRINOLOGY: Approaches to molecular genetic diagnosis in the management of differences/disorders of sex development (DSD): position paper of EU COST Action BM 1303 ‘DSDnet’. European Journal of Endocrinology 2018 179 R197–R206. (10.1530/EJE-18-0256) PMC6182188 30299888

[19] Hughes JJ Alkhunaizi E Kruszka P Pyle LC Grange DK Berger SI Payne KK Masser-Frye D Hu T Christie MR Loss-of-function variants in Ppp1r12A: from isolated sex reversal to holoprosencephaly spectrum and urogenital malformations. American Journal of Human Genetics 2020 106 121–128. (10.1016/j.ajhg.2019.12.004) 31883643 PMC7042489

[20] Thompson JJ Williams CS. Protein phosphatase 2A in the regulation of Wnt signaling, stem cells, and cancer. Genes 2018 9 121. (10.3390/genes9030121) PMC5867842 29495399

